# One-step fabrication of robust superhydrophobic and superoleophilic surfaces with self-cleaning and oil/water separation function

**DOI:** 10.1038/s41598-018-22241-9

**Published:** 2018-03-01

**Authors:** Zhi-hui Zhang, Hu-jun Wang, Yun-hong Liang, Xiu-juan Li, Lu-quan Ren, Zhen-quan Cui, Cheng Luo

**Affiliations:** 1 The Key Laboratory of Bionic Engineering, Ministry of Education, Jilin University, Changchun, 130022 People’s Republic of China; 2 State Key Laboratory of Automotive Simulation and Control, Jilin University, Changchun, 130022 People’s Republic of China

## Abstract

Superhydrophobic surfaces have great potential for application in self-cleaning and oil/water separation. However, the large-scale practical applications of superhydrophobic coating surfaces are impeded by many factors, such as complicated fabrication processes, the use of fluorinated reagents and noxious organic solvents and poor mechanical stability. Herein, we describe the successful preparation of a fluorine-free multifunctional coating without noxious organic solvents that was brushed, dipped or sprayed onto glass slides and stainless-steel meshes as substrates. The obtained multifunctional superhydrophobic and superoleophilic surfaces (MSHOs) demonstrated self-cleaning abilities even when contaminated with or immersed in oil. The superhydrophobic surfaces were robust and maintained their water repellency after being scratched with a knife or abraded with sandpaper for 50 cycles. In addition, stainless-steel meshes sprayed with the coating quickly separated various oil/water mixtures with a high separation efficiency (>93%). Furthermore, the coated mesh maintained a high separation efficiency above 95% over 20 cycles of separation. This simple and effective strategy will inspire the large-scale fabrication of multifunctional surfaces for practical applications in self-cleaning and oil/water separation.

## Introduction

Over the last few decades, superhydrophobic surfaces have attracted considerable attention due to their diverse practical applications, such as self-cleaning^1^, oil/water separation^2^, corrosion resistance^3^, anti-icing^4^, anti-fogging^5^, anti-fouling^6^, anti-bacterial^7^, anti-reflection^8^, and drag reduction^9^. Many functional biological surfaces in nature, including lotus leaves^10^, rose petals^11^, butterfly wings^12^, and water striders^13^, among others^14^, possess unique wettability properties. Studies of these biological surfaces have indicated that the surface roughness originating from unique micro/nanostructures and the surface chemistry are two major factors that affect the surface wettability^15–18^. To date, superhydrophobic surfaces have been developed by a variety of methods, such as sol-gel methods^19^, lithographic processes^20^, casting^21^, electrospinning^22^, chemical vapour deposition (CVD)^23^, chemical etching^24^, dip-coating^25^, and templating^26^. Although these approaches allow for the manufacture of superhydrophobic surfaces, many limitations stemming from the complicated and time-consuming fabrication processes, expensive equipment, and restrictions of substrate materials prevent the large-scale application of most of these methods^27,28^. Thus, low-cost, facile methods for fabricating superhydrophobic surfaces for widespread applications are urgently needed. Brush-coating, dip-coating and spray-coating are typical methods that can meet the abovementioned requirements and facilitate the practical application of superhydrophobic surfaces. However, the mechanical weakness of the micro/nanostructures on the coating surface limits the applications of superhydrophobic coatings^29–31^. To overcome this limitation, many studies have employed an adhesive layer to bond the coating to a substrate and enhance the robustness of the surface. Two strategies are available for producing an adhesive layer. One of the strategies is useful but not perfect because the adhesive and coating need to be separately applied to the substrate^32,33^. This two-step coating strategy is time-consuming, and the thickness of the resulting coating is restricted, which limits the large-scale application of the resulting superhydrophobic surface. Another important strategy is to thoroughly mix the adhesive with the coating and then deposit the mixture on the substrate through a one-step approach^34^. This method can be used to effectively fabricate robust superhydrophobic surfaces and has great potential for large-scale application. Fluorinated reagents with low surface free energies are often applied when constructing superhydrophobic surfaces to improve the hydrophobicity of the material surface^35–38^. However, fluoro-containing compounds are expensive and harmful to the environment and human health^39–41^. Therefore, fluorine-free compounds that are inexpensive and environmentally friendly, such as alkyl silanes and long carbon chain organics, have been developed for the fabrication of superhydrophobic surfaces^42–44^. Furthermore, noxious organic solvents are often used to prepare coating suspensions during the fabrication of superhydrophobic surfaces, which is environmentally hazardous. Thus, environmentally friendly solvents should be used for the large-scale fabrication of superhydrophobic surfaces.

Superhydrophobic surfaces are easily contaminated with oil, causing the loss of their self-cleaning abilities^45^. Manufacturing superamphiphobic surfaces that can repel both water and oil is a promising method for overcoming this disadvantage^46^. However, the applications of superamphiphobic surfaces are limited to specific fields. For example, in the field of machinery, certain parts such as bearings, chains and gears need to be lubricated with oil to reduce friction^32^. In anti-corrosion applications, oil-lubricated metal surfaces can resist rust for long periods by providing a barrier against air and moisture. However, because superamphiphobic surfaces repel oil, they cannot be lubricated by oil. In addition, fluorinated reagents are needed for the fabrication of almost all superamphiphobic surfaces. In recent years, only a few reports have demonstrated the preparation of robust superhydrophobic and superoleophilic surfaces that possess self-cleaning capabilities in both air and oil^32,33^. Furthermore, most of these robust self-cleaning surfaces that function in both air and oil are fabricated through time-consuming two-step methods.

In addition to self-cleaning, another important application of superhydrophobic and superoleophilic surfaces is the separation of oil and water^47–49^. The separation capability of the surface is typically evaluated in terms of the separation efficiency and reusability^50^. Although various strategies for oil/water separation have been developed, several shortcomings still need to be overcome, such as fouling of the surface by oil and poor recyclability^51–53^. These shortcomings lead to poor performance in continuous oil/water separation for practical applications.

**Figure 1:**
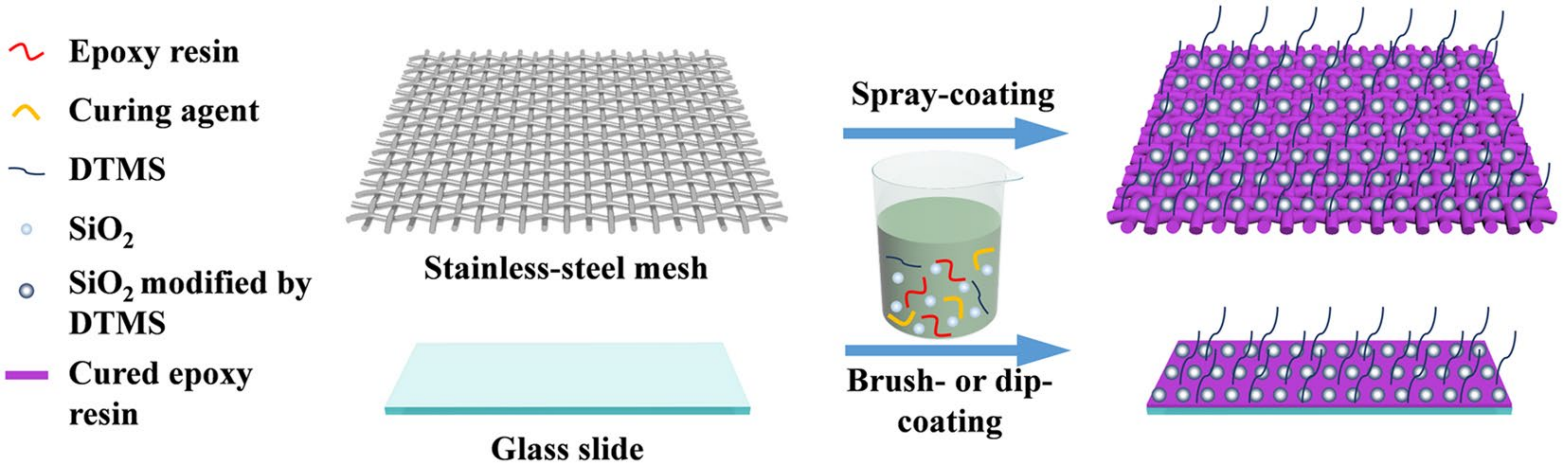
Schematic illustration for the fabrication of MSHOs by one-step deposition methods.

Herein, we report a fluorine-free coating for fabricating robust superhydrophobic and superoleophilic surfaces that possess self-cleaning abilities in both air and oil and can be used in highly effective oil/water separations. This strategy used epoxy resin (ER) as the adhesive, providing the resulting surfaces with microscale roughness and robustness. Silica nanoparticles and dodecyltrimethoxysilane (DTMS) were employed to enhance the nanoscale roughness and reduce the surface free energy, respectively. The as-obtained paint-like suspensions were painted on glass slides and stainless-steel meshes as substrates through brush-coating, dip-coating and spray-coating methods (as illustrated in Fig. 1). The morphologies, chemical compositions, and wettability of the coated surfaces were characterized. Self-cleaning tests in both air and oil demonstrated that the self-cleaning performance of the coated surfaces was maintained, even in oily environments. The coated surfaces were confirmed to be robust through abrasion and knife-scratch tests. Finally, the coated stainless-steel meshes were used to separate various oil/water mixtures, and the obtained separation efficiency was greater than 93%. The coated meshes maintained a high separation efficiency (>95%) with a chloroform/water mixture after 20 repeated separation cycles. Thus, this easily-manufactured, eco-friendly and multifunctional surface with superhydrophobic and superoleophilic properties has great potential for practical application in diverse fields.

## Results and Discussion

### Surface morphologies

**Figure 2:**
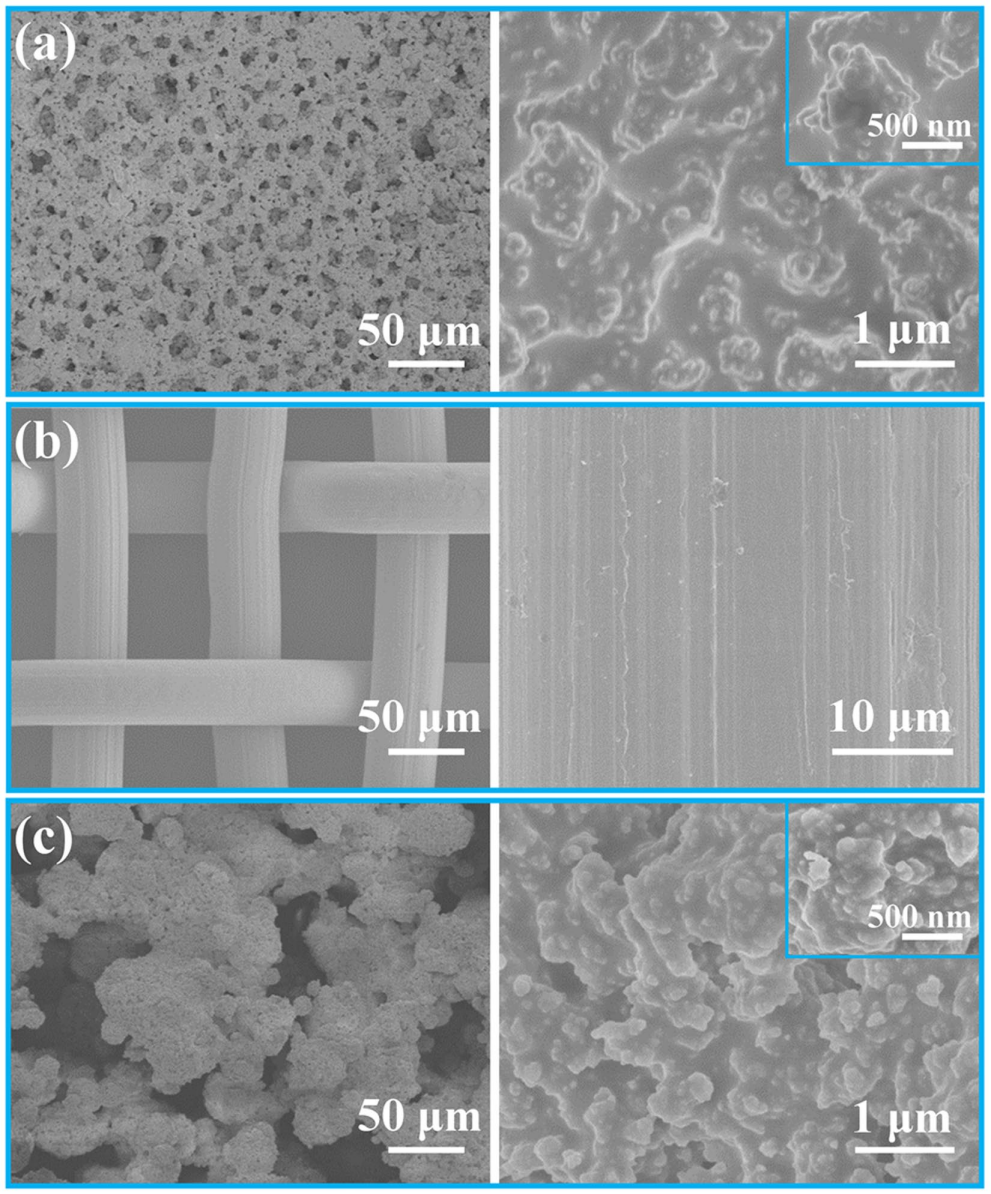
Structural characterization of the coating and stainless-steel mesh. Low-magnification and enlarged FESEM images of (**a**) the brush-coated glass slide, (**b**) the original mesh, and (**c**) the spray-coated mesh.

The surface morphologies of the brush-coated glass slides, pristine stainless-steel meshes and spray-coated meshes were characterized by field emission scanning electron microscopy (FESEM). As shown in Fig. 2a, the coated glass slide exhibited microporous structures, which formed from the rapid evaporation of anhydrous alcohol. As shown in the enlarged SEM image, the coating possessed a rough surface with hierarchical micro/nanoscale structures (Fig. 2a). Meanwhile, numerous nanoscale papillae structures were observed. These hierarchical micro/nanoscale rough structures (HMNRs) played an important role in establishing the superhydrophobicity. Figure 2b depicts a typical image of the untreated stainless-steel mesh, which exhibited a smooth surface. After coating the mesh as described, the steel wires were completely covered by the coating material (Fig. 2c). It should be noted that the presence of micropores could ensure the free passage of oil through the coated mesh. The surface morphology of the coated mesh was different from that of the coated glass slide in low-magnification owing to the difference of coating methods and substrates. Nevertheless, the magnified FESEM image of the coated mesh also showed the presence of HMNRs, which provided an important foundation for establishing the superhydrophobic properties. In addition, the average surface roughness of coatings on glass slides and meshes was measured using a laser scanning confocal microscopy (LSCM). As shown in Supplementary Fig. S1, the average mean square roughness of the coated mesh (Ra = 3.93 μm) was larger than that of the coated glass slide (Ra = 6.78 μm). This is due to the uneven surface of the original meshes and the difference of the coating methods.

### Chemical composition

**Figure 3:**
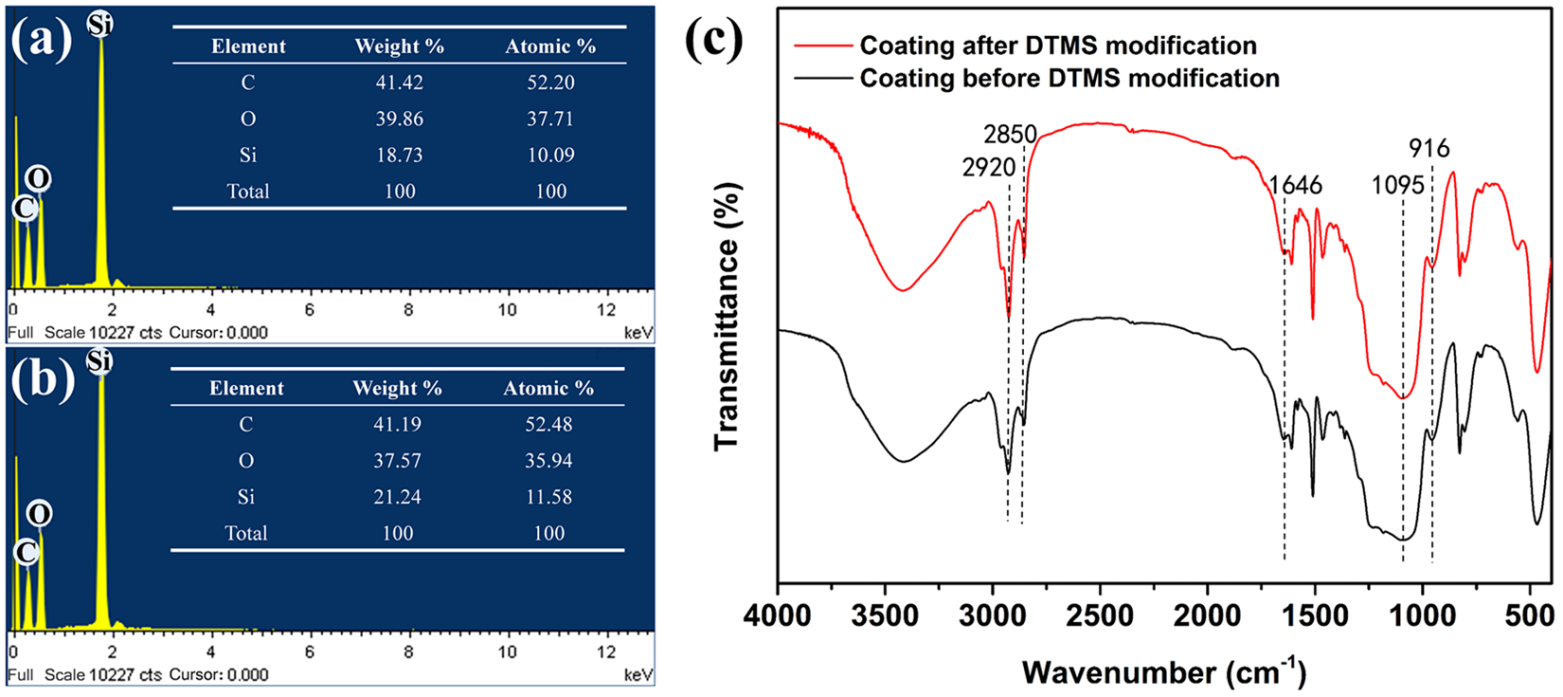
Chemical compositions of the coatings. EDS spectra of the coatings before (**a**) and after (**b**) modification with DTMS, respectively. (**c**) FTIR spectra of the coatings before and after modification with DTMS.

The differences in the chemical compositions of the unmodified hydrophobic coatings and the superhydrophobic coatings modified by DTMS were analysed by energy dispersive spectrometer (EDS) and fourier transform infrared spectrum (FTIR). Figure 3a,b show the EDS spectra of the coatings before and after modification with DTMS, respectively. The atomic ratio of Si increased from 10.09% (Fig. 3a) to 11.58% (Fig. 3b) with the addition of DTMS. As displayed in Fig. 3c, the characteristic peaks of the coating before modification were observed at 916 cm^−1^ for the -CH(O)CH- groups of ER, 1095 cm^−1^ for the Si-O-Si groups of SiO_2_ and 1646 cm^−1^ for the C=O groups of polyamide (PA)^54^. In addition, after modification, the Si-O-Si (1095 cm^−1^), -CH2- (2850 cm^−1^) and -CH3 (2920 cm^−1^) stretching vibrations became stronger than those in the unmodified coating, thereby demonstrating that the coating was successfully modified with DTMS^42,55^.

### Surface wettability

**Figure 4:**
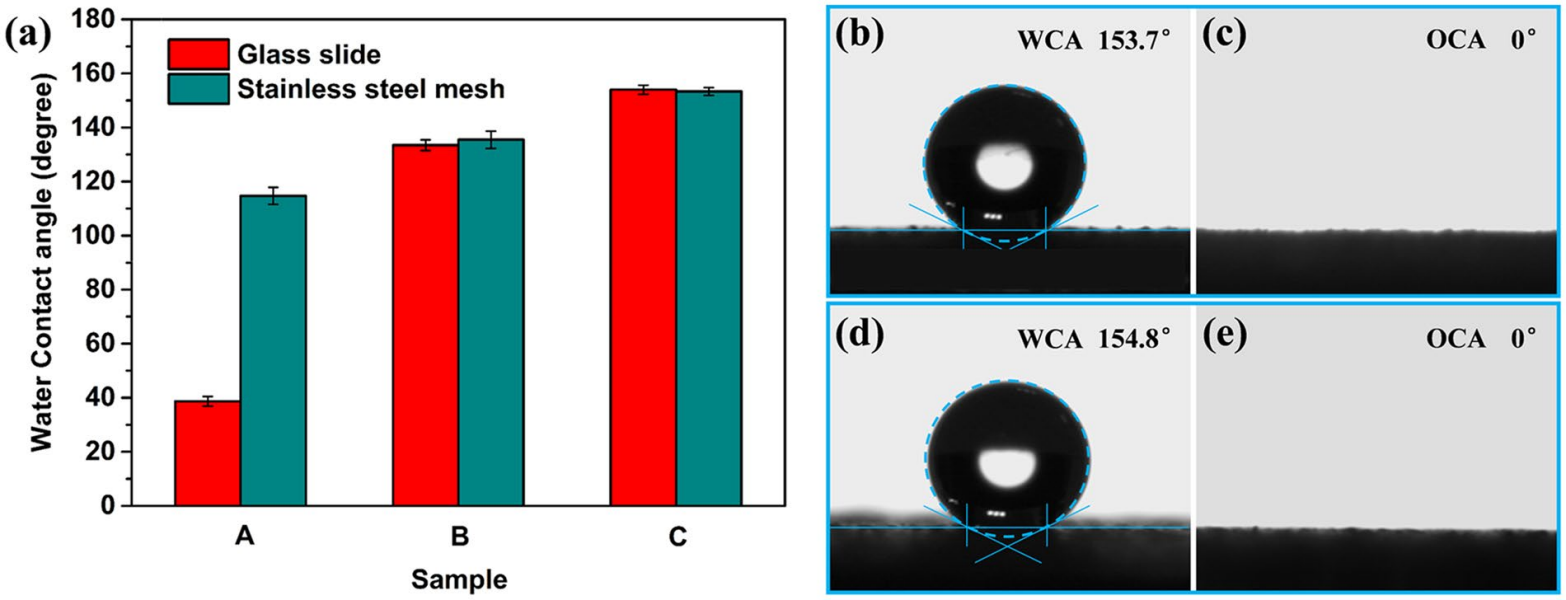
(**a**) The water wettability of A original glass slides and stainless-steel meshes, B coated glass slides and meshes using the coating without DTMS, and C coated glass slides and meshes modified by DTMS. (**b**) and (**c**) are WCA and OCA images of coated glass slides, respectively. (**d**) and (**e**) are WCA and OCA images of coated meshes, respectively.

The wettability of the coated glass slides and coated meshes was characterized by the water contact angle (WCA), oil contact angle (OCA) and water sliding angle (WSA). The effects of the surface micro/nanoscale structures and the chemical compositions on the wettability were also investigated. As shown in Fig. 4a, the original glass slide and mesh exhibited hydrophilic and hydrophobic properties, respectively. After introduction of the coating without DTMS, the hydrophilic glass slide became hydrophobic, with a WCA of 133.5° ± 2°. Meanwhile, the coated mesh became even more hydrophobic (WCA = 135.5° ± 3.2°) than the coated glass slide. Furthermore, the coated glass slides (WCA = 154° ± 1.7°) and meshes (WCA = 153.3° ± 1.4°) modified with DTMS showed superhydrophobic properties and had WSAs below 5°. Thus, the HMNRs of the coating surfaces were not sufficient for imparting superhydrophobic properties on the coated surfaces. However, the low surface free energy of the coating that resulted from modification with DTMS established superhydrophobic properties. In other words, both appropriate HMNRs and chemical compositions were essential for the fabrication of superhydrophobic surfaces. Furthermore, the coated surfaces were not only superhydrophobic but also showed superoleophilic properties, with an OCA of 0° (Fig. 4b,c).

**Figure 5:**
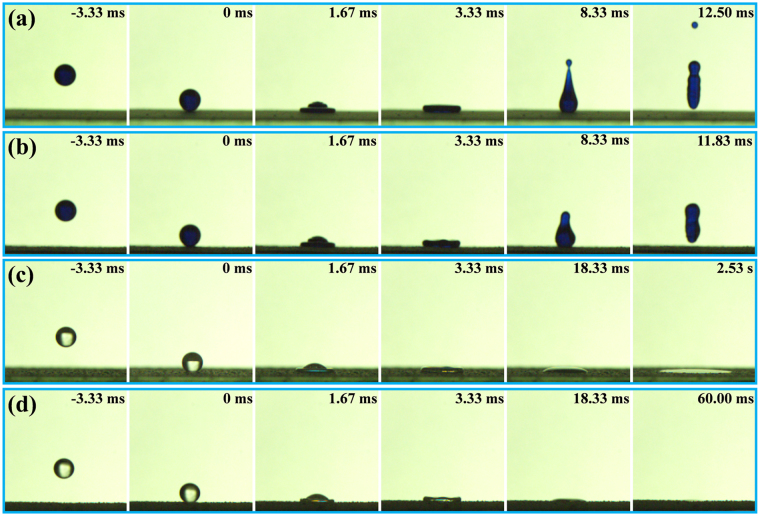
Water bouncing and oil spreading processes on the coated glass slides and stainless-steel meshes. Bouncing dynamics of a water droplet impacting (**a**) a coated glass slide and (**b**) a coated mesh. (**c**) Spreading processes of an oil droplet impacting (a) a coated glass slide and (**d**) a coated mesh.

To further confirm the superhydrophobic and superoleophilic behaviours of the coated surfaces, the bouncing or spreading of water and oil droplets were observed with a high-speed camera. Using a microinjector, water droplets with a volume of approximately 6.7 μL were dropped from a height of 35 mm. The water droplets then impacted the coated glass slide or mesh at a speed of 0.83 m/s. When the water droplet was dropped onto a coated glass slide, it bounced and completely left the coated surface within 12.5 ms (Fig. 5a), and the coated glass slide was not wetted by the water droplet dyed blue with methylene blue. Similarly, the water droplet dropped onto a coated stainless-steel mesh bounced and completely left the coated surface within 11.83 ms, as shown in Fig. 5b. The coated mesh was also not wetted by the coloured water droplet. The observed water-repellent behaviours of the coated glass slide and mesh surfaces further demonstrated the superhydrophobicity of the coated surfaces. In addition, oil dropping tests were carried out with the same parameters as those for the water dropping tests described above. The oil droplets had a volume of approximately 6.7 μL and impacted the coated glass slide or mesh at approximately 0.83 m/s. As shown in Fig. 5c, the oil droplet completely spread out on the coated glass slide within 2.53 s. After impacting the coated mesh, the oil droplet spread out and permeated into the mesh within 60 ms. The rapid spread of the oil droplets on the coated surfaces further illustrated the superoleophilic properties of the coatings. These superhydrophobic and superoleophilic behaviours of the coating surfaces suggested that the coating could be useful for self-cleaning and oil/water separation applications.

### Self-cleaning in air or oil

**Figure 6:**
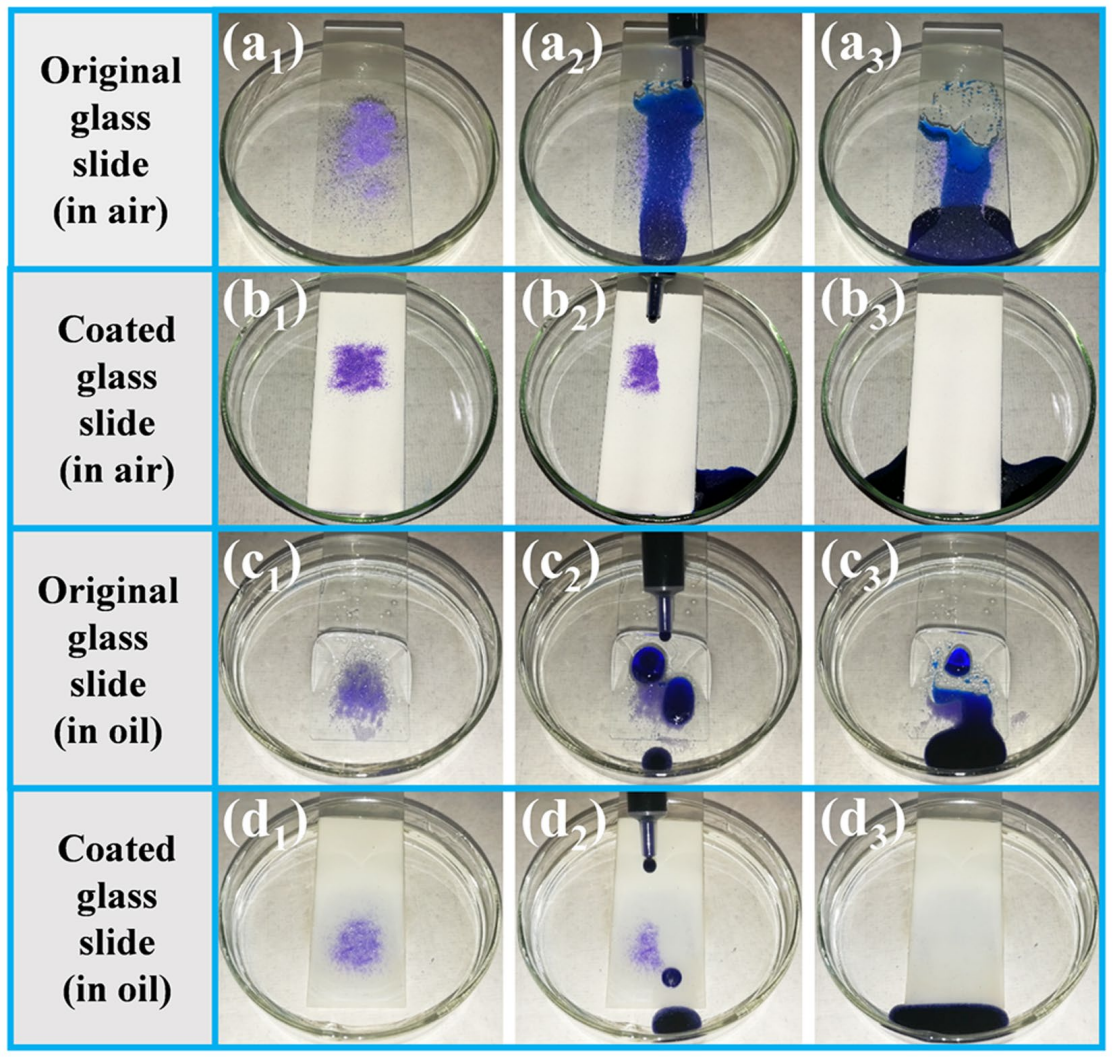
Self-cleaning tests in air or oil. Self-cleaning properties of (**a**_**1**_–**a**_**3**_) the original glass slide and (**b**_**1**_–**b**_**3**_) the coated glass slide in air. Self-cleaning properties of (**c**_**1**_–**c**_**3**_) the original glass slide and (**d**_**1**_–**d**_**3**_) the coated glass slide in oil. When self-cleaning tests were carried out in oil, the glass slides were first completely contaminated with oil and then partially immersed in oil. The tilt angle was approximately 12°.

A series of self-cleaning tests were carried out to demonstrate the self-cleaning properties of the coated surfaces. In these experiments, purple sand was utilized as dirt to aid visualization. For the self-cleaning test in air, the dirt was obviously not removed from the uncoated glass slide, which was wetted and contaminated by the coloured water (Fig. 6a_1_–a_3_). This result indicates that the uncoated glass slide did not have self-cleaning abilities. In contrast, no dirt or dyed water remained on the coated glass slide after the removal of dirt by rolling water droplets, illustrating the excellent water-repelling and self-cleaning properties (Fig. 6b_1_–b_3_ and Supplementary Video S1). For the coated glass slides, air was trapped in the HMNRs instead of water, forming an air layer. This trapped air substantially decreased the contact area between the water droplet and the solid surface. Therefore, water droplets in the Cassie state easily rolled off of the superhydrophobic coated surfaces due to the low water adhesion properties of the surfaces.

**Figure 7:**
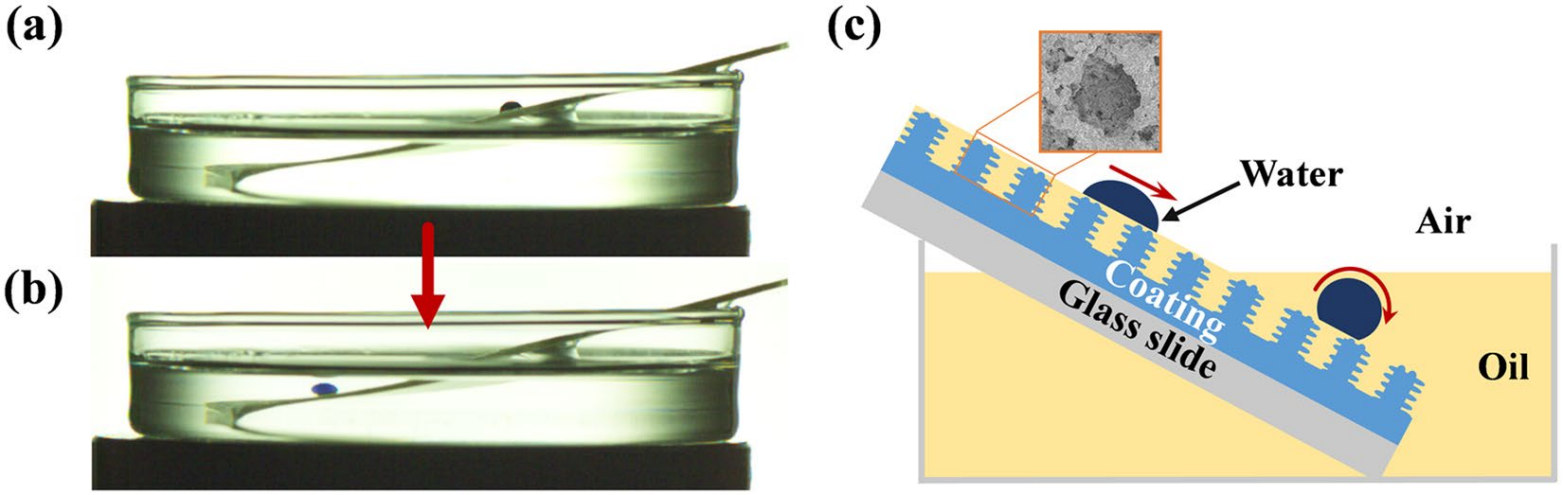
Movement of water droplets on a coated glass slide contaminated with or immersed in oil. (**a**) The water droplet slid on the coated glass slide contaminated by oil and (**b**) rolled on the coated glass slide immersed in oil. (**c**) Schematic of a water droplet moving on an oil-contaminated coated glass slide that is partially immersed in oil.

Most self-cleaning tests of coatings with unique wettability properties were performed in air, not in oil^32^. The changes in the physical and chemical properties of the coated surface after contamination with oil resulted in the weakening or even loss of the self-cleaning abilities. Herein, the self-cleaning properties of the superhydrophobic and superoleophilic coating are demonstrated. Self-cleaning tests were carried out on uncoated and coated glass slides contaminated with or immersed in oil (hexadecane). The pristine glass slide did not show self-cleaning abilities under these conditions (Fig. 6c_1_–c_3_). However, for the coated glass slide contaminated with oil, the oil was locked in the HMNRs of the coating, forming a thin oil film (Fig. 7c). Because the surface tension of oil is lower than that of water, the relatively stable oil film could not be replaced by water. Therefore, a slippery liquid-infused porous surface (SLIPS) that repelled water droplets was formed, and the water droplet could easily slide across the coated surface as a result (Fig. 7a and Supplementary Video S2). Furthermore, the water droplets removed the dirt without wetting the coated glass slide (Fig. 6d_1_–d_3_ and Supplementary Video S3). When the coated glass slides were immersed in oil, the oil penetrated into the HMNRs of the coating. Due to the HMNRs, the low surface free energy of the coated surface and the surrounding oil, the dyed water droplet was spherical and easily rolled off of the tilted surface (Fig. 7b and Supplementary Video S2). No water adhered to the oil-contaminated coated surface that was partially immersed in oil (Fig. 6d_1_–d_3_ and Supplementary Video S3). These results show that the coated surfaces still retained their self-cleaning properties when contaminated with or even immersed in oil. The self-cleaning tests of the coated glass slides were repeated in hexane and silicon oil to further demonstrate the self-cleaning properties. As shown in Supplementary Fig. S2, the water droplets removed the purple sand without staining the coated surfaces, illustrating the excellent self-cleaning properties of the coating. The experimental results show that the multifunctional superhydrophobic and superoleophilic surfaces (MSHOs) with excellent self-cleaning properties in both air and oil possess broad potential for application in complex practical environments, especially oily environments.

### Robustness of coatings

**Figure 8:**
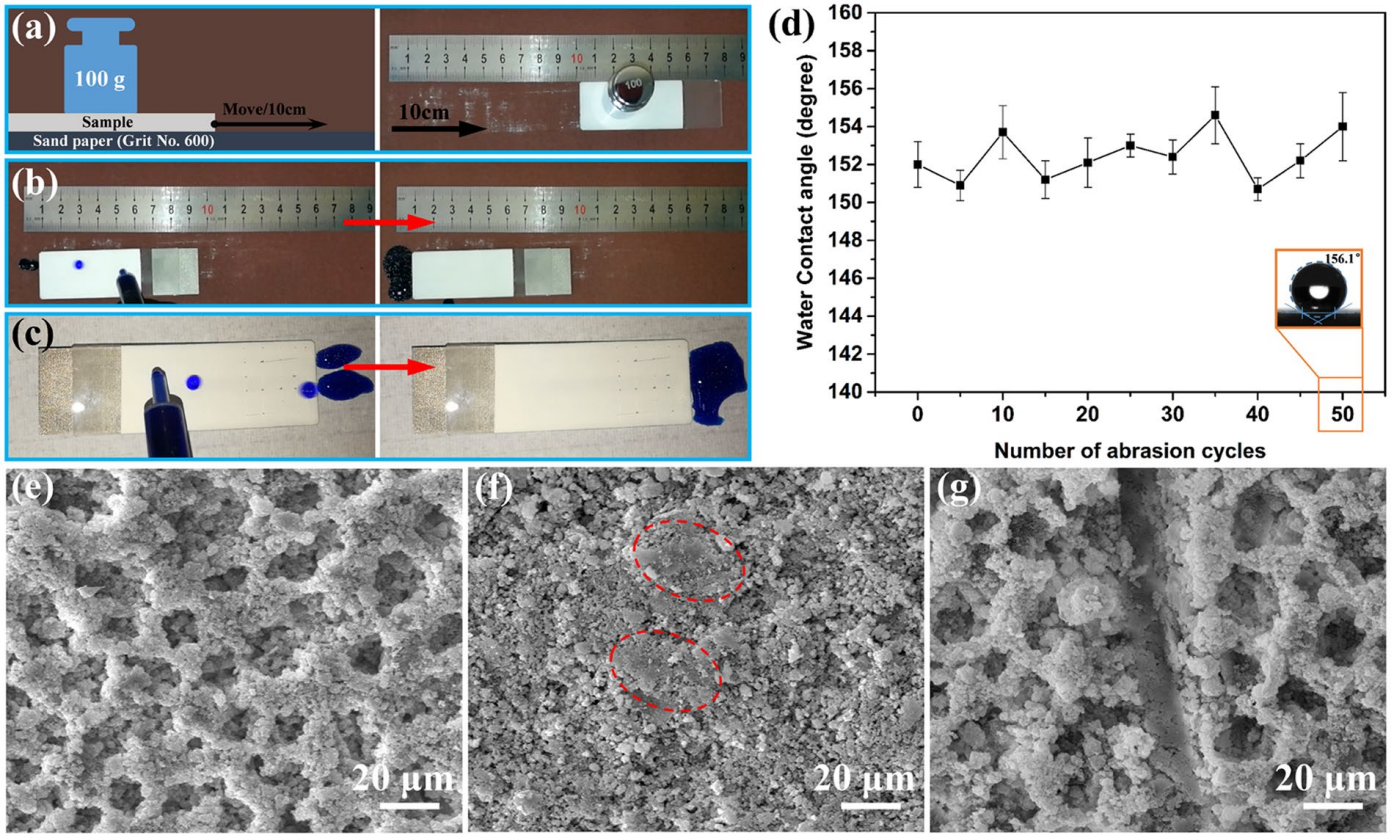
(**a**) Schematic illustration of the sandpaper abrasion test. Water-repellent behaviour of the dip-coated surface after (**b**) abrasion with sandpaper and (**c**) scratching with a knife. (**d**) WCAs of the dip-coated surface after every fifth abrasion cycle. SEM images of the dip-coated surface before (**a**) and after (**b**) abrasion with sandpaper and (**c**) scratching with a knife.

Micro/nanoscale structures are essential for establishing superhydrophobic properties in coatings. However, these structures are mechanically weak and easily worn, which limits the widespread application of superhydrophobic coatings. The use of an adhesive to bond the coating to the substrate can enhance the robustness of the coating. However, if the adhesive and coating must be individually painted on the substrate, the coating process becomes relatively complicated, which will affect the efficiency of the large-scale production. Here, we added ER and PA as adhesives to the paint-like suspensions to improve the poor robustness of the resulting superhydrophobic coatings and simplify the coating processes. Then, sandpaper abrasion and cross-cut scratching tests were carried out to examine the robustness of the obtained coatings. A schematic illustration of the sandpaper abrasion test is shown in Fig. 8a. The dip-coated surface maintained its water repellency after abrasion with sandpaper (Fig. 8b and Supplementary Video S4). As shown in Fig. 8d, the WCA varied between 150° and 156° in the 50 abrasion cycles, indicating that the superhydrophobicity of the coatings was not easily destroyed under mechanical abrasion. Figure 8f shows only a small part of the coating surface becomes smoother (the red dashed frames) and most of the coating surface possesses micro/nanoscale roughness structures after 50 abrasion cycles. This phenomenon could be explained that only the top layer of the micropores on the coating surface was removed due to the robustness of the coating and the micro/nanoscale roughness structures in the micropores were exposed (Fig. 8e and Fig. 8f). In addition, some micro/nanoscale roughness structures could be produced by sandpaper wearing. The cross-cut scratching test showed that the water droplets rolled off from the coating surfaces which were scratched by a knife without wetting the surfaces (Fig. 8c and Supplementary Video S5). Although the scalpel knife truly damaged the roughness structures along the scratch due to its sharpness and hardness (Fig. 8g and Supplementary Video S5), it did not significantly influence the excellent water repellency of the coating in function, which was extremely important for practical applications. The as-prepared coating surfaces were concluded to be robust based on the results of the sandpaper abrasion and cross-cut scratching tests. The good adhesion and mechanical properties of the cured ER might be responsible for the good robustness. Since the coating could be painted on the substrate in a one-step coating process and the resulting superhydrophobic surface was robust, the coating might be widely used in the large-scale manufacture of superhydrophobic surfaces.

### Separation of oil/water mixtures

**Figure 9:**
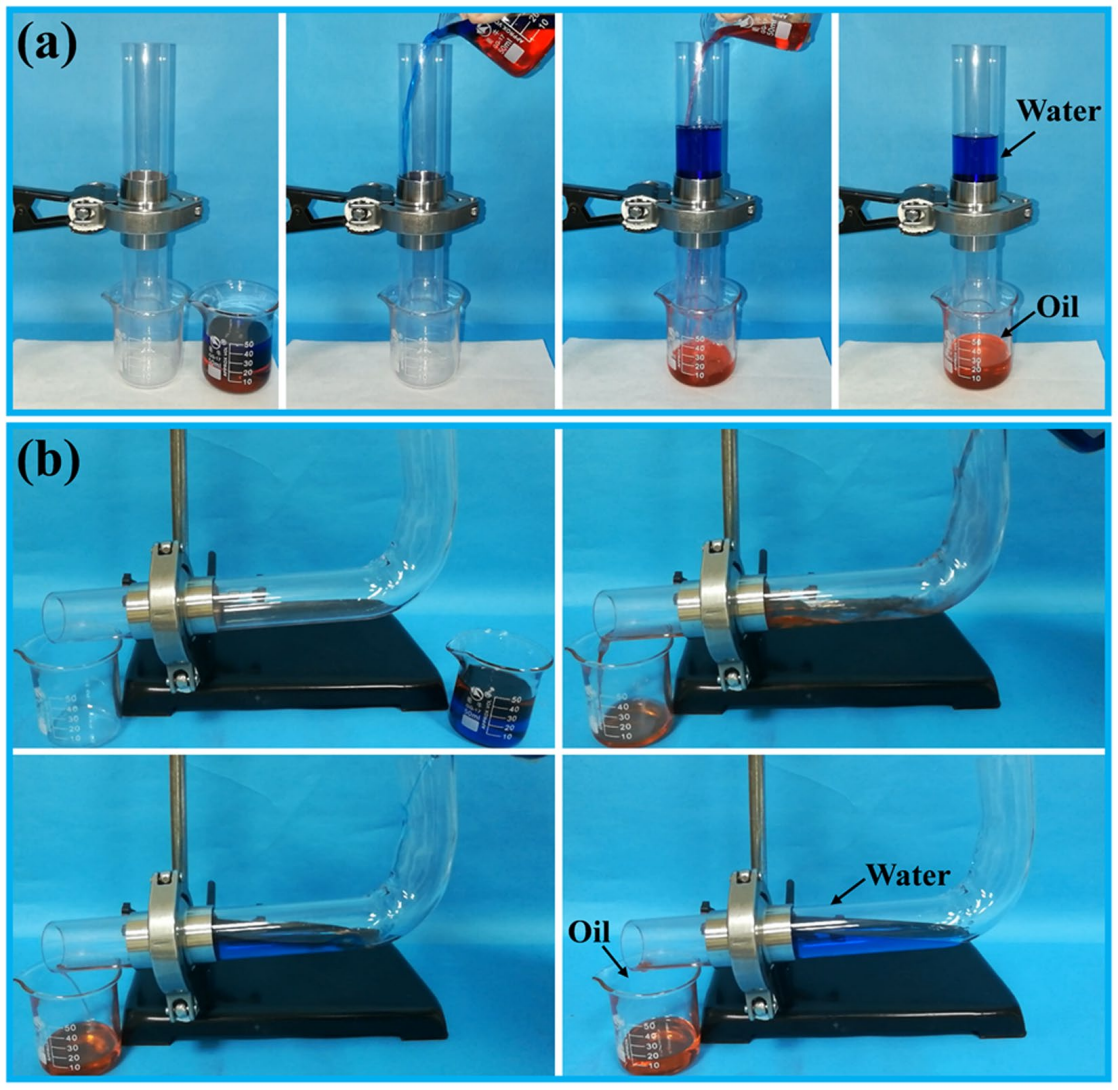
Separation of a (**a**) a heavy oil/water mixture and (**b**) a light oil/water mixture by using the coated mesh. The water was dyed blue with methylene blue, and the oil was coloured red with sudan III.

Superhydrophobic and superoleophilic porous materials have potential for use in the separation of oil/water mixtures. Here, oil/water separation tests were performed to investigate the potential of the coated stainless-steel mesh for application in oil/water separation. Figure 9a shows the separation procedure for a mixture containing 20 mL of heavy oil (chloroform) and 30 mL of water. The chloroform quickly permeated through the coated mesh under only gravity and was collected in the beaker below, whereas the water was retained in the glass tube above the coated mesh (Supplementary Video S6). These experimental results reveal the ability of the coated mesh to efficiently separate heavy oil/water mixtures. During the separation of light oil/water mixtures, if the mixture was quickly poured into the same separation device as that shown in Fig. 9a, some oil eventually floated on the collected water, resulting in a low separation efficiency. This phenomenon occurred because the permeate flux of the coated mesh was within a certain range. Hence, a mixture containing 20 mL of light oil (hexane) and 30 mL of water was separated in the experimental setup shown in Fig. 9b. The hexane rapidly passed through the coated mesh and was collected in the beaker underneath, while the water was completely retained above the coated mesh (Supplementary Video S7).

**Figure 10:**
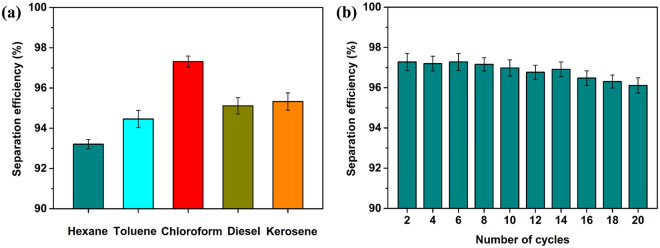
(**a**) Separation efficiencies of the coated mesh with various oil/water mixtures. (**b**) Separation efficiency of the coated mesh with a chloroform/water mixture during repeated experiments.

The separation efficiency is often used to evaluate the ability to perform oil/water separation and is calculated according to the equation *η* = *(m*_1_*/m*_0_) × 100%, where *m*_0_ and *m*_1_ are the mass of oil before and after separation, respectively^50^. As shown in Fig. 10a, the separation efficiency of the coated mesh with a variety of oil/water mixtures was above 93%. The most efficient separation by the coated mesh was accomplished with the chloroform/water mixture, which gave a separation efficiency above 97%. A small gap remained between the practical separation efficiency and 100%, which was mainly attributed to the volatilization of a small amount of oil, while another portion of the oil was absorbed by the coated mesh or adhered to the separation device. Due to the combined effects of the volatility, density and viscosity of the oils, the separation efficiency of the coated meshes with different oils varied slightly. Moreover, the reusability of the coated mesh was evaluated because this property is important in practical applications. As shown in Fig. 10b, the coated mesh maintained a separation efficiency above 95% with a chloroform/water mixture after 20 separation cycles, which indicated that the mesh had excellent reusability. Therefore the as-prepared coated mesh was proven to be useful for continuous and efficient oil/water separation.

## Conclusions

In summary, MSHOs were easily fabricated through one-step coating methods of brush-coating, dip-coating and spray-coating. The obtained surfaces maintained their unique wettability after being abraded with sandpaper or scratched with a knife, thus exhibiting excellent robustness due to the good adhesion and mechanical properties of the added ER. The multifunctional surfaces showed excellent self-cleaning performance in both air and oil and were successfully used to quickly separate oil/water mixtures with a high separation efficiency. After 20 separation cycles, the separation efficiency remained above 95%, indicating the excellent reusability of the coated mesh. The facile fabrication strategy of the MSHOs with excellent robustness is believed to promote the application of self-cleaning surfaces in harsh and oily environments and also be useful for continuous and efficient oil/water separations.

## Methods

### Materials

Stainless-steel meshes with a pore diameter of ≈ 50 μm were purchased from Changzhou Zanbang Screen Trading Co., Ltd. ER E51 and PA 650 were obtained from Hangzhou Wuhui Port Adhesive Co., Ltd. Silica (approximately 30 nm in diameter) modified with γ-aminopropyltriethoxysilane (KH-550) was purchased from Zhoushan Mingri Nanomateraials Co., Ltd. DTMS was obtained from Macklin. Hexadecane, sudan III and methylene blue were purchased from Tianjin Guangfu Technology Development Co., Ltd. Silicon oil with a viscosity of 20 cSt was obtained from Dow Corning. Anhydrous ethanol, toluene, chloroform and hexane were purchased from Beijing Chemical Works. Diesel and kerosene were produced by SINOPEC. All reagents were used as received without further purification.

### Preparation of the coating

Silica nanoparticles (20 g) were placed in 100 mL of anhydrous ethanol at room temperature, and the solution was stirred for 30 min to ensure good dispersion. To enhance the robustness and hydrophobicity of the resulting coatings, 10 g of ER as an adhesive and 4 mL of DTMS as a modifier were added to the solution, which was then stirred for 60 min. After that, 6 g of PA was added to the solution as a curing agent, and the paint-like suspension was stirred for 30 min. The glass slides and stainless-steel meshes used as substrates were successively rinsed with anhydrous ethanol and distilled water. After drying, the coating solution was deposited on the glass slides by the brush-coating and dip-coating methods and on the meshes by spraying. MSHOs were thereafter obtained after solidification of the coatings for 24 h at room temperature.

### Instruments and characterization

The surface morphology was characterized FESEM (Hitachi, S-4800) at an accelerating voltage of 2 kV. Measurements of surface roughness were carried out using a LSCM (Olympus, Ols 3000). The chemical constituents and their contents were identified with the help of EDS (Oxford, X-Max^N^ 150). FTIR (Bruker, Equinox 55) measurements were performed to examine the various chemical bonds. The dropping of water and oil droplets on the coated surfaces and the movement of oil droplets on coated glass slides contaminated with or immersed in oil were monitored using a high-speed camera (Vision Research, Phantom V711) at 6000 frame s^−1^ and 1000 frame s^−1^, respectively. The contact angles and sliding angles were measured at ambient temperature using an optical contact angle meter (OCA 20, Dataphysics). Water and oil droplets (5 μL) were carefully dropped onto the surfaces, and the average contact angle was obtained by measuring different positions on the same surface.

### Self-cleaning tests in air and oil

For the self-cleaning test in air, a coated glass slide was placed in a clean petri dish at an angle of approximately 12°. Purple sand (with sizes in the range of 50–120 μm) was place on top of the coated glass slide to acting as artificial dirt. Water was dyed blue with methylene blue for visual differentiation. Then, dyed water droplets were dropped onto the coated glass slide one at a time. In the following experiments, we determined the self-cleaning function of coated glass slides contaminated with oil or immersed in oil. An oil-contaminated coated glass slide was partially immersed in oil at a tilt angle of approximately 12°, and dirt was layered on the coated surface in both the oil and air. Dyed water droplets were dropped onto the coated surface one at a time, and the removal of dirt from the coated surface via the rolling water droplets was recorded.

### Sandpaper abrasion and scratch tests

Sandpaper abrasion and scratch tests were carried out to examine the robustness of the superhydrophobic and superoleophilic coatings. For the sandpaper abrasion test, a dip-coated glass slide was placed face-down on sandpaper (standard sandpaper, grit no. 600), and a 100 g weight was placed on top of it. The coated glass slide was transversely moved by 10 cm, which was defined as 1 abrasion cycle. A total of 50 abrasion cycles were performed, and the average WCA was measured after every 5th abrasion cycle. In addition, a scratch test was performed using a knife to longitudinally and transversely scratch the dip-coated surface, after which the water-repellent properties of the scratched surface were examined.

### Oil/water separation

The coated meshes (45 mm × 45 mm) were fixed between two stainless-steel holders. Two glass tubes with diameters of 24 mm were attached to the holders. A series of oil/water mixtures and organic solvent/water mixtures, including kerosene, diesel, hexane, toluene and chloroform, were separated. The separation device was installed at an angle of 90° when separating mixtures of heavy oil and water and at 5° when separating mixtures of light oil and water. The chosen angles allowed the oil to be fully accessible to the coated mesh, which improved the separation efficiency of the light oil/water mixtures. To ensure clear visual differentiation, the oil in the mixture was dyed red with sudan III, and the water was dyed blue with methylene blue. During the separation processes, the mixture of oil/water was poured in the glass tube from above. The only driving force for the separation was gravity.

### Data Availability

All data generated or analysed during this study are included in this published article (and its Supplementary Information files).

## Electronic supplementary material

Supplementary Information

Video S1

Video S2

Video S3

Video S4

Video S5

Video S6

Video S7
