# Supplementary material for: One-step fabrication of robust superhydrophobic and superoleophilic surfaces with self-cleaning and oil/water separation function
Source: Sci Rep. 2018 Mar 1;8:3869. doi: 10.1038/s41598-018-22241-9 (PMC5832778; doi:10.1038/s41598-018-22241-9)
Supplement: Supplementary file 1 — Supplementary Information [file 41598_2018_22241_MOESM1_ESM.pdf]

One-step fabrication of robust superhydrophobic and superoleophilic surfaces with self-cleaning and oil/water separation function

Zhi-hui Zhang,[1,2] Hu-jun Wang,[1] Yun-hong Liang,*[1,2] Xiu-juan Li,[1] Lu-quan Ren,[1] Zhen-quan Cui[1] and Cheng Luo[1]

[1]The Key Laboratory of Bionic Engineering, Ministry of Education, Jilin University, Changchun 130022, People's Republic of China.

[2]State Key Laboratory of Automotive Simulation and Control, Jilin University, Changchun 130022, People's Republic of China.

*Corresponding Author: Yunhong Liang, E-mail: liangyunhong@jlu.edu.cn

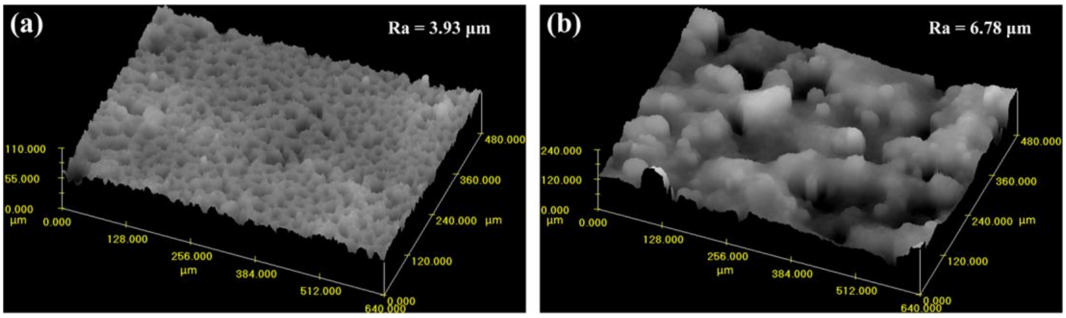

Figure S1. LSCM images of the coating on (a) the glass slide and (b) the stainless-steel mesh.

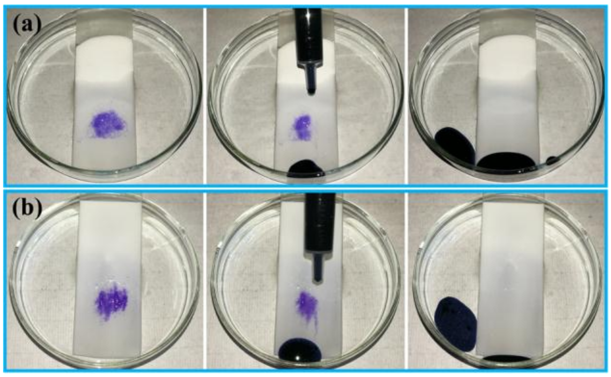

Figure S2. Self-cleaning tests of coated glass slides contaminated with or immersed in (a) hexane and (b) silicon oil with a viscosity of 20 cSt. The coated glass slides were first completely contaminated with oil and then partially immersed in the oil. The tilt angle was approximately 12°.

Video S1. Self-cleaning test of a coated glass slide in air.

Video S2. Movement of a water droplet on a coated glass slide that was first contaminated with oil and then partially immersed in oil.

Video S3. Self-cleaning test of a coated glass slide in oil.

Video S4. Maintained water repellency of the coated surface after abrasion with sandpaper.

Video S5. Maintained water repellency of the coated surface after damage by scratching with a knife.

Video S6. Separation of a heavy oil/water mixture.

Video S7. Separation of a light oil/water mixture.
